# The Challenges of Fetal Atrial Septal Interventions in Hypoplastic Left Heart Syndrome

**DOI:** 10.1016/j.jaccas.2026.108617

**Published:** 2026-06-17

**Authors:** Michał Gałeczka, Roland Fiszer, Michał Hawranek

**Affiliations:** a Department of Congenital Heart Diseases and Pediatric Cardiology, Medical University of Silesia, Silesian Center for Heart Diseases, Zabrze, Poland; b 3rd Department of Cardiology, School of Medical Sciences in Zabrze, Silesian Center for Heart Diseases, Medical University of Silesia, Katowice, Poland

**Keywords:** congenital heart defect, cyanotic heart disease, left-sided catheterization, treatment, ultrasound

**Figure:**
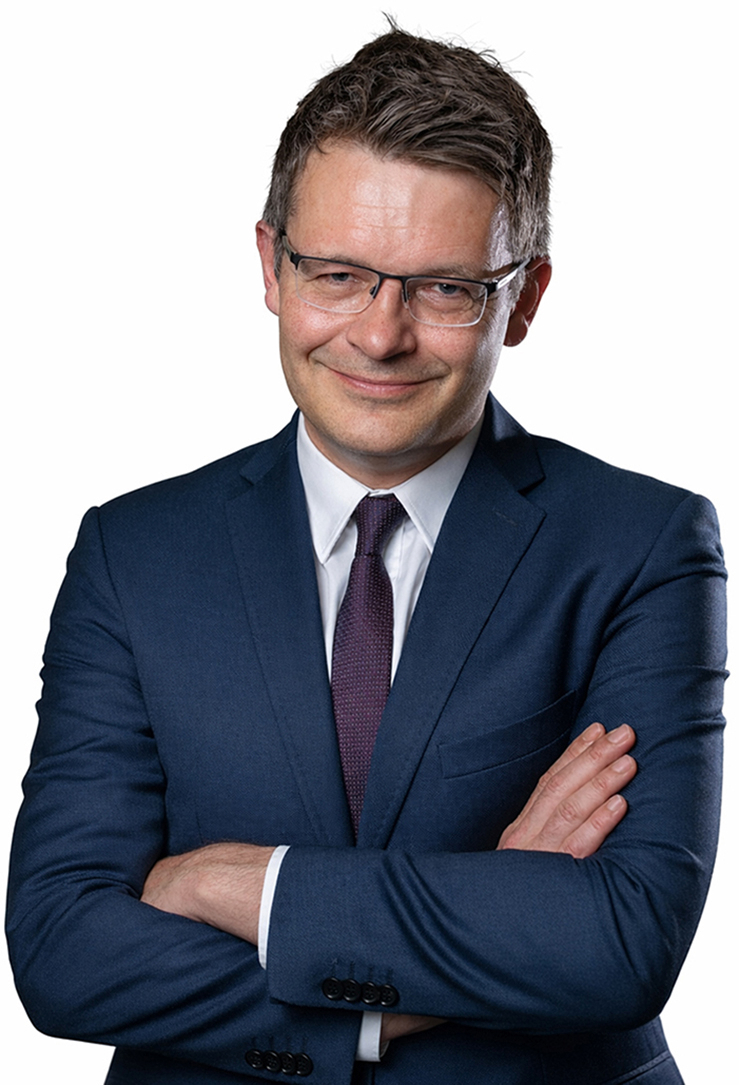

Hypoplastic left heart syndrome (HLHS) remains one of the most formidable congenital heart defects. It is characterized by severe hypoplasia or atresia of left-sided structures, including the mitral and aortic valves, the left atrium and ventricle, and the aorta. Currently, no curative intervention exists; management is restricted to either a 3-stage palliative Fontan pathway or neonatal heart transplantation.^1^^,^^2^ Although transplant-free survival to adulthood is approximately 60%, patients with HLHS face a significantly more guarded prognosis compared with those with other single-ventricle morphologies.^2^ Furthermore, Fontan physiology is frequently burdened by diminished quality of life and a high incidence of long-term morbidities.^2^ Progression toward various phenotypes of circulatory failure is common; although transplantation remains a rescue option, this cohort faces a 10-year post–transplant survival rate of only 68%—the lowest among all congenital heart disease subgroups.^3^

HLHS with a restrictive atrial septum (RAS) represents the most critical phenotype of this defect. The resulting pulmonary vascular congestion can induce prenatal remodeling of the pulmonary vasculature and parenchyma, often culminating in pulmonary lymphangiectasia. Postnatally, RAS precipitates profound neonatal instability; consequently, mortality in this subgroup is exceptionally high, often exceeding 50% within the first weeks of life.^4^

To mitigate these outcomes, fetal atrial septal interventions (FASIs)—such as balloon septoplasty and stenting—were developed in the early 2000s to decompress the left atrium and prevent irreversible lung damage, often referred to as “nutmeg lung.”^5^^,^^6^ Historically, FASI is the prenatal successor to the landmark Raskind procedure of the 1960s. Just as the Raskind atrioseptostomy revolutionized the management of transposition of the great arteries, FASIs have catalyzed a new era in fetal interventional cardiology.^7^

Despite its potential, FASI remains an exceptionally rare procedure with limited longitudinal data regarding its feasibility and long-term outcomes. Although technical success rates have reached 80% with no maternal mortality reported to date, the complication profile remains significant, occurring in up to 65% of cases. These include hemopericardium requiring drainage (40%-50%), bradycardia—as seen in the current case—and fetal demise (10%-20%).^8^, ^9^, ^10^, ^11^ Among newborns who undergo successful FASIs, <60% ultimately proceed to surgical palliation, with overall survival to hospital discharge estimated at 35% to 48%.^8^^,^^9^ Nevertheless, successful FASIs offer undeniable clinical benefits, including a reduced incidence of Cesarean delivery and a decreased requirement for emergent postnatal resuscitation.^9^

The procedure is performed at only a few specialized centers worldwide, requiring a multidisciplinary team capable of maneuvering within a fetal heart that measures only 30 mm in diameter at 30 weeks of gestation.^8^^,^^9^^,^^11^, ^12^, ^13^ Although fetal aortic valvuloplasty has shown promise in promoting biventricular circulation, the definitive long-term benefit of FASIs remain to be fully established.^9^

Specific technical choices within FASIs also warrant scrutiny. In this issue of *JACC: Case Reports*, Scharnreitner et al^10^ describe the first-in-human use of an everolimus-eluting stent (DES) for fetal atrial septal stenting in HLHS with an intact atrial septum instead of a standard bare-metal stent to mitigate in-stent obstruction from endothelial overgrowth.^10^ This approach successfully resulted in permanent decompression and the resolution of pleural effusions.^10^ This strategy mirrors protocols in ductus arteriosus stenting, where DES has reduced luminal loss without increasing infection risks.^14^

Mechanistically, mTOR inhibitors such as sirolimus and everolimus have been administered to fetuses primarily to manage rhabdomyoma progression and capillary-lymphatic-venous malformations.^15^^,^^16^ Although sirolimus is frequently the first-line agent, everolimus offers distinct advantages, including a shorter half-life and more stable plasma concentrations.^17^ However, systemic mTOR inhibition is not without risk to the maternal-fetal unit, with complications such as fetal growth restriction documented.^17^ Notably, the localized serum concentration of the inhibitor eluted from a DES is expected to be several orders of magnitude lower than that of systemic therapy, likely rendering the risk of systemic immunosuppression negligible in this setting.

However, a primary concern with a DES in this context is the contraindication of antiplatelet therapy. Although a DES is the gold standard for adults, the risk of stent thrombosis makes dual antiplatelet therapy mandatory for at least 1 month.^18^ The clinical profile of an adult with atherosclerosis is fundamentally different from that of a fetus. Aspirin is strictly contraindicated in the third trimester because of the risk of premature closure of the ductus arteriosus—a vital structure in HLHS—and reduced amniotic fluid and neonatal intracranial bleeding. Consequently, the balance between potential thrombosis risk without antiplatelet therapy and the benefit of reducing in-stent overgrowth remains uncertain and warrants further investigation.

Regarding long-term prognosis, although Fontan eligibility guidelines have become more liberal,^19^ patients with HLHS-RAS often struggle to meet the criteria for a “good” Fontan candidate, even after successful FASI.

This leads to an unavoidable dilemma. An 18-year survival rate of 60% for HLHS is a modern medical miracle; however, many survivors face “Fontan failure,” liver disease, and cognitive challenges.^2^ The ethical debate reflects the ongoing challenge of balancing improved survival with long-term morbidity and quality of life in this complex population. Procedures like FASIs, supported by evolving techniques such as the use of pressure wires to monitor gradients, continue to push these survival percentages higher. This pursuit requires an extraordinary clinical effort that deserves recognition. Importantly, the present report represents a valuable proof-of-concept, demonstrating the feasibility of drug-eluting stent use in the fetal setting and providing a basis for future investigation.

## Funding Support and Author Disclosures

The authors have reported that they have no relationships relevant to the contents of this paper to disclose.
